# Synthesis and catalytic properties of a series of cobalt porphyrins as cytochrome P450 model: the effect of substituents on the catalytic activity

**DOI:** 10.1007/s10847-012-0205-x

**Published:** 2012-07-05

**Authors:** Bingcheng Hu, Chengguo Sun, Quanzhi Deng, Zuliang Liu

**Affiliations:** School of Chemical Engineering, Nanjing University of Science and Technology, Nanjing, 210094 Jiangsu China

**Keywords:** Deuteroporphyrin, Oxidation of toluene, Cobalt porphyrin, Hemim, Catalyst

## Abstract

A series of cobalt porphyrins derived from hemin was prepared as cytochrome P450 models. Effects of substituents at the cobalt deuteroporphyrin-propionate side chains are investigated in oxidation of toluene with air to benzaldehyde and benzyl alcohol without the use of solvent and sacrificial co-reductant. The catalytic activity of cobalt porphyrins depends on the type of substituents. When the electron-withdrawing groups like –Cl, –Br, were introduced into the double propionate side chains, they can increase the catalyst stability and selectivity to benzaldehyde. In comparison with these electron-withdrawing groups, the electron-donor groups, such as –CH_3_, –S–S– and –NH_2_ groups, can improve their catalytic activities. Moreover, the electron-donor group containing an unpaired electron (such as –S–S–, –NH_2_) is benefit for improving its catalytic efficiency and promoting the electron delivery. It can be concluded that the double propionate side chains in the deuteroporphyrin complex may participate in oxidation process and effect electron transfer from the high-valent metalloporphyrin species to the substrate.

## Introduction

During the past decades, introduction of peripheral substituents on the porphyrin macrocycle has been used as an effective strategy to increase the stability of metalloporphyrins against oxidative degradation and develop new porphyrin catalysts with enhanced catalytic activity for hydroxylation of hydrocarbon [1–4]. The full understanding of the relationship between the catalytic activity and the structure of metalloporphyrins turns out to be an important issue in designing catalysts for industrial applications. As a result, a lot of efforts have been invested to synthesize multi-substituted porphyrins, trying to elucidate the role of the substituents and the changes of catalytic performances of catalysts [5, 6]. Indeed, the several major studies have proved that halogenated, nitrified metalloporphyrins are more active as catalysts and more resistant to oxidative degradation than their unsubstituted analogues [7, 8]. Although the wide studies have focused on the effect of substituents of *meso*-tetraphenylporphyrins on their catalytic activity, the relationship between the structure of porphyrin and the effect of peripheral substituents, especially at the propionate side chains, is still unclear, because synthetic *meso*-tetraphenylporphyrin analogues deviate dramatically from natural porphyrins [9].

**Scheme 1:**
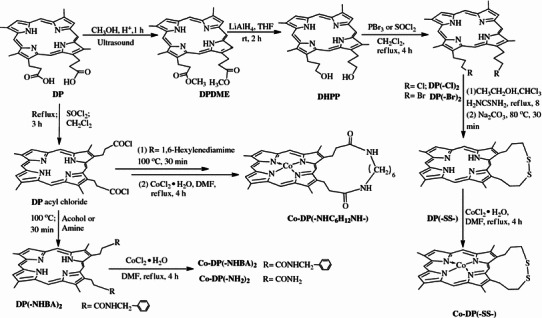
Schematic structure of the Cobalt deuteroporphyrin derivatives in this study

In the previous work, the oxidation of cyclohexane with air catalyzed by hemin derivatives has been investigated and the propionate side chains are important factors influencing the catalytic efficiency of the metallodeuteroporphyrin dimethyl ester [10, 11]. For a systematic study, in this paper we have prepared a series of cobalt deuteroporphyrin derivatives (Co-DPs) by modification of the hemin-propionate side chains (Scheme 1) and investigated the effects of peripheral substituents at the deuteroporphyrin-propionate side chains in the oxidation of toluene under the conditions of 150 °C and 0.7 MPa using air as oxygen donor. To our knowledge, this is the first time to investigate the effect of substituents of hemin derivatives on catalyzing the oxidation of toluene.

## Experimental

### General methods

All reagents and solvent used were of analytical grate. Deuteroporphyrin (DP) was synthesized and purified according to our recent report [12]. Tetrahydrofuran (THF) was dried by distillation from sodium and benzophenone. ^1^H NMR was recorded on a Bruker 500 MHz spectrometers with tetramethylsilane (TMS) as an internal standard. LC–MS/MS (ESI) mass spectra were recorded on a Finnigan TSQ Quantum ultra AM mass spectrometer in conjunction with a Finnigan Surveyor^®^ LC system comprising of an HPLC with a built-in degasser, autosampler and PDA detector (Thermo Electron Corporation, San Jose, CA, USA). Column effluent was introduced into the electrospray chamber with a 0.1 mm internal diameter fused silica capillary. Electrospray ionization spray voltage was 3,800 V in positive mode. Nitrogen was used as sheath gas at 30 psi and as the auxiliary gas at 10 psi, and argon as the collision gas at 1.2 mTorr, with the capillary temperature 270 °C. Analytes were introduced into the electrospray source via an Thermo DB-C18 2.1 mm × 150 mm column at a flow rate of 20 μL/min (methanol/water = 80/20). The UV–Vis spectra were measured by a Shimadzu UV-240 spectrophotometer. The absorption changes versus time at the λ_max_ of Co-DPs have been used to determine the extent of degradation of catalyst in reaction conditions. IR spectra were obtained by a Perkin–Elmer 681 instrument with KBr optics. GC–MS analyses were performed on a Thermo Trace DSQ mass spectrometer. The electrochemical determination was tested in a Chen Hua CHI 660 Electrochemical Workstation. Redox potentials of the compounds (10^−3^ mol/L) in dried DMF containing 0.1 mol/L tetrabutylammonium perchlorate (TBAP) as supporting electrolyte were determined at room temperature by cyclic voltammetry using platinum–carbon electrode, Pt and SCE as working, counter and reference electrodes, respectively.

### Preparation of deuteroporphyrin dimethyl ester DP(–OCH_3_)_2_

To a stirred mixture of DP(–OCH_3_)_2_ (0.20 g, 0.37 mmol) in dry THF (30 mL) was added a solution of LiAlH_4_ (0.050 g, 1.32 mmol) in dry THF (10 mL). The mixture was stirred at rt for 2 h after which ethyl acetate (3 mL) was added. The reaction mixture was further stirred for another 10 min and adjusted the PH of solution to 6.0 by diluted hydrochloric acid. The organic layer was extracted with CH_2_Cl_2_, dried over sodium sulfate. Column chromatography (silica gel/dichloromethane:methanol = 10:1) followed by usual workups gave the product. Yield: 0.13 g (0.27 mmol, 73 %). m.p: >250 °C; 1HNMR (500 MHz, DMSO-d_6_) δ/ppm : = −4.01 (s, 2H, –NH); 2.38 (m, 4H); 3.48 (dd, 4H, *J* = 7.5 Hz); 3.61, 3.66, 3.73, 3.77 (4s, 12H); 4.15 (t, *J* = 7.5 Hz, 4H); 4. 81 (t, 2H); 9.33, 9.35(2s, 2H); 10.29, 10.30, 10.32, 10.33(4s, 4H); IR (KBr, cm^−1^): 3450 (m, OH), 3320 (m, NH), 2932 (m, CH), 1639 (w, C=C), 1043 (m), 1061 (m), 1001 (m), 845 (m), 742 (m), 534 (s); ESI^+^-MS (*m*/*z*): 483.04 [M + H]^+^. Anal. Calcd for C_30_H_34_N_4_O_2_: C, 74.66; H, 7.10; N, 11.61; Found: C, 74.92; H, 7.60; N, 11.53.

### Preparation of 2,7,12,18-tetram ethyl-13,17-di(3-hydroxypropyl) porphyrin DHPP

To a stirred mixture of DP(–OCH_3_)_2_ (0.20 g, 0.37 mmol) in dry THF (30 mL) was added a solution of LiAlH_4_ (0.050 g, 1.32 mmol) in dry THF (10 mL). The mixture was stirred at rt for 2 h after which ethyl acetate (3 mL) was added. The reaction mixture was further stirred for another 10 min and adjusted the PH of solution to 6.0 by diluted hydrochloric acid. The organic layer was extracted with CH_2_Cl_2_, dried over sodium sulfate. Column chromatography (silica gel/dichloromethane:methanol = 10:1) followed by usual workups gave the product. Yield: 0.13 g (0.27 mmol, 73 %). m.p: >250 °C; 1HNMR (500 MHz, DMSO-d_6_) δ/ppm : = −4.01 (s, 2H, –NH); 2.38 (m, 4H); 3.48 (dd, 4H, *J* = 7.5 Hz); 3.61, 3.66, 3.73, 3.77 (4s, 12H); 4.15 (t, *J* = 7.5 Hz, 4H); 4. 81 (t, 2H); 9.33, 9.35(2s, 2H); 10.29, 10.30, 10.32, 10.33(4s, 4H); IR (KBr, cm^−1^): 3450 (m, OH), 3320 (m, NH), 2932 (m, CH), 1639 (w, C=C), 1043 (m), 1061 (m), 1001 (m), 845 (m), 742 (m), 534 (s); ESI^+^ -MS (*m*/*z*): 483.04 [M + H]^+^. Anal. Calcd for C_30_H_34_N_4_O_2_: C, 74.66; H, 7.10; N, 11.61; Found: C, 74.92; H, 7.60; N, 11.53.

### Synthesis of 2,7,12,18-tetram ethyl-13,17-di(bromic or chloric-propyl) porphyrin DP(-Br)_2_, DP(-Cl)_2_

A solution of DHPP (0.20 g, 0.41 mmol) in 40 mL of dichloromethane was treated at 39 °C with 0.4 mL of PBr_3_ or SOCl_2_ in 5 mL of CH_2_Cl_2_ under the condition of stirring for 4 h. After completion, the reaction mixture was washed with water, extracted with CH_2_Cl_2_. The solution was concentrated and purified by silica gel chromatography (CH_2_Cl_2_:EtOAc = 10:1) to give 0.21 g (0.35 mmol, 84 %) of DP(–Br)_2_ and 0.17 g (0.33 mmol, 81 %) of DP(-Cl)_2_ as black solids, respectively.

DP(–Br)_2_, m.p: >250 °C; ^1^H NMR (500 MHz, CDCl_3_) δ/ppm = −3.87 (s, 2H), 2.85–2.88 (t, *J* = 6.5 Hz, 4 H), 3.71–3.78 (m, 16 H), 4.29 (t, *J* = 6.5 Hz, 4 H), 9.12, 9.13 (2s, 2 H), 10.08, 10.12, 10.19, 10.24 (4s, 4H). ESI^+^-MS (*m*/*z*): 606.85 [M + H]^+^, Anal. Calcd for C_30_H_32_Br_2_N_4_: C, 59.22; H, 5.30; Br, 26.27; N, 9.21. Found: C, 59.71; H, 5.44; N, 9.70.

DP(-Cl)_2_, m.p: >250 °C; ^1^H NMR (500 MHz, CDCl_3_) δ/ppm = −3.86 (s, 2H), 2.75 (t, *J* = 7.5 Hz, 4 H), 3.62–3.84 (m, 16 H), 4.26 (t, *J* = 7.5 Hz, 4 H), 9.10 (s, 2 H), 10.03, 10.07, 10.14, 10.16 (4s, 4H). ESI^+^-MS (*m*/*z*): 519.07 [M + H]^+^, Anal. Calcd for C_30_H_32_Cl_2_N_4_: C, 69.36; H, 6.21; N, 10.78. Found: C, 69.11; H, 6.69; N, 10.97.

### Synthesis of 2,7,12,18-tetram ethyl-13,17-di(3-disulfidepropyl) porphyrin DP(–SS–)

To a solution of thiourea (0.60 g) in ethanol (50 mL) were added a solution of DP(-Br)_2_ (0.50 g) in CHCl_3_ (25 mL). The mixture was stirred magnetically under reflux condition for 8 h. the process of reaction was monitored by TLC until the DP(-Br)_2_ was consumed. Then, the saturated Na_2_CO_3_ solution was added to the mixture until the pH was 9.0. After stirring at 80 °C for 30 min, the mixture was diluted with CH_2_Cl_2_ (60 mL) and then washed with H_2_O (100 mL). The organic layers were decanted, combined, dried, over NaSO_4_, filtered and concentrated to yield the crude product, which was further purified by silica gel chromatography using CH_2_Cl_2_ as an eluent to provide the desired product in 79 % (0.33 g) yield. m.p: >250 °C; ^1^H NMR (500 MHz, CDCl_3_) δ/ppm = −2.03 (s, 2 H), 2.76 (s, 4 H), 3.07 (t, *J* = 7.5 Hz, 4 H), 3.62, 3.65 (2s, 6 H), 3.73–3.75 (2s, 6 H), 4.32–4.35 (m, 4 H), 9.24, 9.26 (2s, 2 H), 10.53 (s, 1 H), 10.58–10.59 (2s, 2 H), 11.61 (s, 1 H). IR (KBr, cm^−1^): 3693 (m), 2925 (m), 1739 (s), 1554 (m), 1224 (m), 887 (m), 848 (m), 788 (m); ESI^+^-MS (*m*/*z*): 512.97 [M + H]^+^, Anal. Calcd for C_30_H_32_N_4_S_2_: C, 70.27; H, 6.29; N,10.93. Found: C, 70.37; H, 6.21; N, 11.23.

### General procedure for the preparation of DP(–NHBA)_2_, DP(–NHC_6_H_12_NH–) and DP(–NH_2_)_2_

To a solution of deuteroporphyrin (0.20 g, 0.39 mmol) in dry CH_2_Cl_2_ (30 mL) was added a solution (0.2 mL) of SOCl_2_, which dissolved in CH_2_Cl_2_ (5 mL). Then, the reaction mixture was stirred under reflux for 3 h. After the reaction was complete, CH_2_Cl_2_ was evaporated, the excess amine (benzylamine, 1,6-hexylenediamime or NH_3_·H_2_O) dissolved in *N*,*N*-dimethylformamide 10 mL was added and heated at 100 °C for 30 min. At the end of the reaction, the mixture solution was poured into distilled water. The residue was filtered, dried in vacuum and purified by silica gel column chromatography with CH_2_Cl_2_/ethyl acetate (5:2) for DP(–NHBA)_2_ (0.21 g, 0.31 mmol, 78 %) and with CH_2_Cl_2_/CH_3_OH (10:1) for DP(–NHC_6_H_12_NH–) (0.11 g, 0.19 mmol, 48 %) and DP(–NH_2_)_2_ (0.13 g, 0.26 mmol, 66 %). DP(–NHBA)_2_: m.p: >300 °C. ^1^H NMR (500 MHz, DMSO-d_6_): δ/ppm = −3.96 (s, 2 H, NH), 3.60, 3.64, 3.75, 3.78 (4s, 12 H), 4.11–4.13 (d, 4 H,), 4.40 (s, 1 H), 6.61, 6.74 (2s, 2 H), 8.38 (s, 2 H), 9.36–9.37 (s, 2 H), 10.30, 10.35, 10.41 (3s, 4 H); IR (KBr, cm^−1^): 3294 (m, N–H), 2917 (w, C–H), 1633 (s, C=O), 1548 (m), 1453 (m), 1374 (m), 1295 (w), 1266 (w), 1195 (m), 1227 (s), 1109 (m), 1028 (w), 967 (m), 833 (w), 720 (s), 694 (m); ESI^+^-MS (*m*/*z*): 689.21 [M + H]^+^. Anal. Calcd for C_44_H_44_O_2_N_6_: C, 76.72; H, 6.44; N, 12.20. Found:C, 76.33; H, 6.67; N, 12.68.

DP(–NHC_6_H_12_NH–): m.p: >300 °C. ^1^H NMR (500 MHz, DMSO-d_6_): δ/ppm = −4.04 (s, 2 H, NH), 1.72–1.88 (m, 8 H), 2.15–2.28 (m, 4 H), 3.62, 3.66 (2s, 6 H), 3.73–3.77 (2s, 6 H), 4.35 (s, 2 H,), 9.33, 9.35 (s, 2 H), 10.30 (s, 1 H), 10.32(s, 1 H), 10.55 (2s, 2 H). IR (KBr, cm^−1^): 3306 (m, NH), 2925 (s), 1633 (s, C=O), 1544 (m), 1439 (m), 1375 (w), 1228 (w), 1107 (m), 1035 (w), 840 (w). ESI^+^-MS (*m*/*z*): 591.17 [M + H]^+^. Anal. Calcd for C_36_H_42_N_6_O_2_: C, 73.19; H, 7.17; N, 14.23. Found: C, 72.49; H, 7.33; N, 14.72.

DP(-NH_2_)_2_: m.p: >250 °C. ^1^H NMR (500 MHz, DMSO-d_6_): δ/ppm = −4.06 (s, 2 H, NH), 3.03 (t, *J* = 7.5 Hz, 4 H), 3.63, 3.66, 3.73, 3.76 (4s, 12 H, 2-, 7-, 12-, 18-CH_3_), 4.33 (t, *J* = 7.5 Hz, 4 H), 6.89, 6.93 (2s, 4 H), 7.50 (s, 2 H), 9.33 (s, 2 H), 10.28, 10.31, 10.35, 10.40 (4s, 4 H). IR (KBr, cm^−1^): 3309 (m, NH), 3171 (m, NH), 2917 (w), 1651 (s, C=O), 1406 (m), 1282 (m), 1229 (w), 1135 (w), 1107 (m), 971 (m), 842 (w), 803 (s), 728 (m), 666 (w). ESI^+^-MS (*m*/*z*): 509.10 [M + H]^+^. Anal. Calcd for C_30_H_32_O_2_N_6_: C, 70.84; H, 6.34; N, 16.52. Found:C, 71.35; H, 6.61; N, 15.97.

### General procedure for the metallation of porphyrin

Cobalt porphyrins were synthesized by the reaction of free base porphyrin (0.10 g) with CoCl_2_·6H_2_O (0.30 g) in DMF (30 mL) to afford high yields of more than 95 %. The reaction condition was under reflux for 4 h. After the completion of the reaction, the reaction was quenched with water (300 mL) and the mixture was extracted with CH_2_Cl_2_ (100 mL). The organic layer was dried with magnesium sulfate and the solvents were evaporated. The crude compound was purified by chromatography on silica gel using CH_2_Cl_2_ and methanol (10:1–10:3) as the mobile phase. All products were analyzed and characterized by LC–MS to indicate a high state of purity. (a) Co-DP(–OCH_3_)_2_, ESI^+^-MS (*m*/*z*): 595.08 [M]^+^. UV–Vis (CH_2_Cl_2_), λ_max_ (nm):390, 512, 546; (b) Co-DP(-Br)_2_, ESI^+^-MS (*m*/*z*): 664.89 [M]^+^. UV–Vis (CH_2_Cl_2_), λ_max_ (nm):395, 499, 547; (c) Co-DP(–Cl)_2_, ESI^+^-MS (*m*/*z*): 575.15 [M]^+^. UV–Vis (CH_2_Cl_2_), λ_max_ (nm): 390, 517, 548; (d) Co-DP(–NHBA)_2_, ESI^+^-MS (*m*/*z*): 745.14 [M]^+^. UV–Vis (CH_2_Cl_2_), λ_max_ (nm): 415, 527, 558; (e) Co-DP(–NHC_6_H_12_NH–), ESI^+^-MS (*m*/*z*): 647.11 [M]^+^. UV–Vis (CH_2_Cl_2_), λ_max_ (nm): 412, 526, 556; (f) Co-DP(–NH_2_)_2_, ESI + -MS (*m*/*z*): 565.03 [M]^+^. UV–Vis (DMF), λ_max_ (nm): 414, 525, 558; (g) Co-DP(**-**SS**-**)_2_, ESI^+^-MS (*m*/*z*): 568.93 [M]^+^. UV–Vis (CH_2_Cl_2_), λ_max_ (nm): 391, 521, 547.

### Oxidation of toluene catalyzed by Cobalt porphyrins with air

Oxidation of toluene was carried out in a stainless steel autoclave of 1 L volume equipped with a mechanical stirrer, an internal thermocouple and cooling coils for regulating the reaction temperature. In a typical experiment, organic phase consisting of 300 mL toluene and 3.0 × 10^−5^ mol/L of metalloporphyrin were mixed with 50 mL nitrobenzene as an internal standard substance. The experiment was performed at 150 °C under the air pressure of 0.7 MPa. The reaction mixture was sampled by an “on-line” means every 30 min until the yield decreased markedly. The samples and the final products were analyzed by GC–MS.

## Results and discussion

### Stability of catalyst

**Fig. 1:**
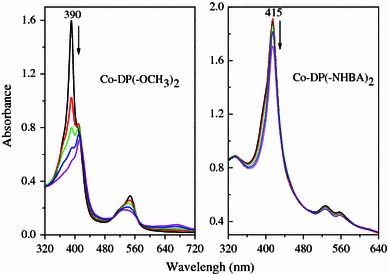
UV–Vis spectrum of a solution of Co-DP(–OCH_3_)_2_ and Co-DP(–NHBA)_2_ in CH_2_Cl_2_ and their changes upon addition of *m*-CPBA with 2 min time intervals up to 8 min

Generally, porphyrin macrocycles are subject to oxidation degradation in strong oxidizing medium [2, 13, 14]. The stability of cobalt metalloporphyrins has been studied in the presence of *m*-CPBA in dichloromethane solvent and UV–Vis spectroscopy has been used to determine the extent of degradation of catalyst. In this reaction, we have used catalyst and *m*-CPBA in 1:200 molar ratios. The changes in the UV–Vis spectra of a solution of Co-DP(–OCH_3_)_2_ in dichloromethane at various time intervals after the addition of *m*-CPBA have been shown in Fig. 1. The results indicate that approximately 78.5 % of Co-DP(–OCH_3_)_2_ has been degraded 8 min after the addition of *m*-CPBA. Under the same conditions, 13.3 % of Co-DP(–NHBA)_2_ has been degraded (Fig. 1). The extent of degradation of other cobalt porphyrins, which was measured through the change in absorbance at the Soret band λ_max_ of metalloporphyrins, has been shown in Table 1. The results indicated the stability of Co-DPs in the order Co-DP(–NHBA)_2_ > Co-DP(–Br)_2_ > Co-DP(–NHC_6_H_12_NH–) > Co-DP(–SS–) > Co-DP(–Cl)_2_ > Co-DP(–NH_2_)_2_ > Co-DP(–OCH_3_)_2_. It is observed that with the exception of Co-DP(–NH–C_6_H_12_NH–) and Co-DP(–SS–), there is an approximate linear relationship between the stability of the cobalt porphyrins and the electronic effect of substituents at the propionate side chains. The Co-DP(–NH_2_)_2_ and Co-DP(–OCH_3_)_2_ with the electron-donor groups are less stable than Co-DP(–Br)_2_ and Co-DP(–Cl)_2_ bearing with electron-withdrawing groups. Interestingly, although the group electronegativity of –NHC_6_H_12_NH– and –SS– was lower than that of –NH_2_ and –OCH_3_, Co-DP(–NHC_6_H_12_NH–) and Co-DP(–SS–) are more stable than Co-DP(–NH_2_)_2_ and Co-DP(–OCH_3_)_2_. Apparently, the groups at the propionate side chains of Co-DP(–NHC_6_H_12_NH–) and Co-DP(–SS–) form the closed ring that contributes to metalloporphyrins stability toward oxidative degradation.

**Table 1:** Degradation of Co-DPs in the presence of *m*-CPBA at room temperature

Co-porphyrin	Soret band (nm)	Degradation (%)	Time (min)
Co-DP(–OCH_3_)_2_	390	78.5	8
Co-DP(–NH_2_)_2_	414^a^	56.8	8
Co-DP(–Cl)_2_	390	43.7	8
Co-DP(–SS–)	391	40.0	8
Co-DP(–NHC_6_H_12_NH–)	412	28.6	8
Co-DP(–Br)_2_	395	23.1	8
Co-DP(–NHBA)_2_	415	13.3	8

The molar ratio of catalyst: oxidation is 1:200 in CH_2_Cl_2_

^a^The Co-DP(-NH_2_)_2_ is dissolved in DMF

### Investigation on the catalytic activity of cobalt porphyrins

Synthetic metalloporphyrins with various groups on the phenyl ring have been employed to elucidate the effect of substituent properties on their catalytic activity in oxidation of hydrocarbon compounds [4, 15, 16]. These investigations have shed light on how substituent groups induce changes in the catalytic activity of metalloporphyrins. It is well known that the catalytic activity of the synthetic metalloporphyrin complexes depend on the degree of electron-withdrawing substituents at the phenyl ring. On the basis of these considerations, a series of Co-DPs were selected as catalysts in the oxidation of toluene with air as oxygen donor under the conditions of 150 °C and 0.7 MPa. Table 2 summarizes the data obtained from our catalytic experiments. It may be seen that the selected Co-DPs appeared suitable catalysts for the oxidation of toluene under the chosen conditions. For the cobalt porphyrins with different substituents at the hemin-propionate side chains, the main products are benzaldehyde and benzyl alcohol. As shown in Table 2, the reaction yield varies with the change of the substituents, and the molar ratios of benzaldehyde to benzyl alcohol changes obviously. Based on the total yield of benzaldehyde and benzyl alcohol, it is concluded that the order of catalytic activity of Co-DPs for the oxidation of toluene is as follows: Co-DP(–SS–) > Co-DP(–NH_2_)_2_ > Co-DP(–OCH_3_)_2_ > Co-DP(–NHBA)_2_ > Co-DP(–NHC_6_H_12_NH–) > Co-DP(–Cl)_2_ > Co-DP(–Br)_2_.

**Table 2:** Oxidation of toluene with air catalyzed by various Co-DPs and Half-wave potential data for the metal-centered one-electron reduction of Co-DPs

Cobalt porphyrins	Benzaldehyde yield (%)	Benzyl alcohol yield (%)	Reaction time (h)^a^	E_pa1_ (mV)	E_pc1_ (mV)	Half-wave reduction potential E_1/2_ (mV)^b^
Co-DP(–SS–)	11.6	4.3	5.0	−877.8	−695.4	−786.6
Co-DP(–NH_2_)_2_	10.2	2.9	4.0	−830.8	−591.8	−711.3
Co-DP(–OCH_3_)_2_	10.1	1.6	5.0	−913.2	−694.2	−803.7
Co-DP(–NHBA)_2_	9.4	1.1	6.0	−884.6	−655.2	−768.4
Co-DP(–NH–C_6_H_12_NH–)	8.8	0.5	5.0	−872.6	−664.0	−768.3
Co-DP(–Cl)_2_	1.9	0.1	6.0	−880.0	−677.6	−778.8
Co-DP(–Br)_2_	1.3	–	6.0	−915.2	−650.0	−782.6

*Reaction conditions*: Toluene 300 mL, Co-DPs, Pressure 0.7 MPa, temperature 150 °C

^a^Time is defined as the reaction time until the yield of benzaldehyde and benzyl alcohol reaches the maximum

^b^ *Conditions*: DMF, Co-DPs = 10^−3^ mol/L, TBAB = 0.1 mol/L, scan rate 100 mV/s

To our surprise, the order of catalytic activity disfavors the conclusion that observed the effect of electron-withdrawing substituents at *meso*-postion of tetraphenylporphyrin, which suggests that the double propionate side chains play a special role in the oxidation of toluene. Generally, several factors are considered in the discussion of the correlation between the structure of metalloporphyrins and their catalytic activity in oxidation processes: catalyst stability, electron effects of substituent, selectivity of oxidation products and reduction potential.

Comparison of the results obtained for their stability (Table 1) with those of their activity quoted in Table 2 indicates that the order of stability is nearly opposite to the order of catalytic activity with the exception of Co-DP(–SS–). In the case of Co-DP(–Br)_2_ and Co-DP(–Cl)_2_ with electron-withdrawing groups at the propionate side chains, the yield of benzaldehyde and benzyl alcohol is lower than other catalysts with electron-donor substituents, such as Co-DP(–OCH_3_)_2_, Co-DP(–NH_2_)_2_. It is interesting to note that the effect of substituents was inconsistent with the substituent effects of *meso*-tetraphenylporphyrins. However, the electron-withdrawing substituents at the propionate side chains obviously can improve their stability in the process of oxidative degradation process.

We also found that with electron-donor groups at the propionate side chains among the four cobalt porphyrins, Co-DP(–NHC_6_H_12_NH-), Co-DP(–SS–), Co-DP(–NH_2_)_2_ and Co-DP(–OCH_3_), the best activity for toluene oxide was obtained with Co-DP(–SS–). Co-DP(–NH_2_)_2_ was also more efficient than Co-DP(–OCH_3_), in spite of the high electronegativity of –NH_2_ group in the former compared with the –OCH_3_ of the latter. Namely, our results suggest that the groups at the propionate side chains play an important role in the investigated reaction and they participate in the reaction and partially contribute to tuning of the electron density of the activated hemin species [17]. The –SS– and –NH_2_ groups have the unpaired electrons that are prone to bind the cobalt in porphyrin core by forming the intermolecular interaction in the reaction. This coordination mode would be responsible for the high values of yield for benzaldehyde observed in the Co-DP(–SS–) and Co-DP(–NH_2_)_2_ systems. Such a conclusion proposed in this study is reminiscent of the mechanism proposed by Idemori et al. [18] in the Mn(III) aminophenylporphyrins-catalyzed oxidation of cyclohexane with iodosylbenzene and iodobenzene diacetate.

As shown in Table 2, it has been found that in case of Co-DPs, introduction of electron-donor substituents at the propionate side chains increases the selectivity of benzyl alcohol and the order of ratio of benzaldehyde to benzyl alcohol is opposite to the order of catalytic activity. The low ratio of benzaldehyde to benzyl alcohol was observed in the oxidation of toluene catalyzed by Co-DP(–SS–) and Co-DP(–NH_2_)_2_. This phenomenon may be attributed to the fact that the –SS–or –NH_2_ groups with unpaired electrons can affect the porphyrin–cation radical complexes on the mechanism of electron transfer. It should be noted that the yield of benzyl alcohol is negligible when the –Br group is introduced at the propionate side chains.

**Fig. 2:**
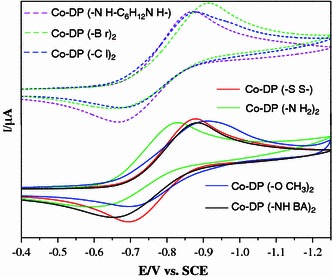
Cyclic voltammograms of Co-DPs (10^−3^ mol/L) in dried DMF containing TBAP (0.1 mol/L) at room temperature (scan rate 100 mV/s)

Furthermore, according to the reported literature, the catalytic activity of metalloporphyrins has the linear relationship with their reduction potential [4, 15]. Figure 2 shows the cyclic voltammograms for these Co-DPs. All of them have exhibited an irreversible wave corresponding to the Co(III)/Co(II) reduction. Table 2 shows the half-wave potentials (E_1/2_) for the one-electron reduction of Co-DPs. Obviously, the half-wave potentials (E_1/2_) for the first reduction of Co-DP(–OCH_3_), Co-DP(–NH_2_)_2_ and Co-DP(–Br)_2_ corresponding to the Co(III)/Co(II) couple were located, respectively, at E_1/2_ = −803.7, −711.3 and −728.6 mV versus SCE in DMF. There is no linear relationship between the catalytic efficiency and the Co(III)/Co(II) potential. However, if we compare the Co-DP(–Br)_2_ with Co-DP(–Cl)_2_, the catalytic activity increases with the increase of the redox potential of cobalt porphyrin. It is important to point out that this single linear relation just exists in the same character of elements or functional groups, for example, –Cl and –Br in the same column of periodic table of elements. For the whole cobalt porphyrins bearing the various substituents, the catalytic efficiency does not correlate with the Co(III)/Co(II) reduction potential. Thus, we conclude that the substitution of propionate side chains act as double roles both improving the stable of structure and affecting the electron transfer. Generally, in the catalytic cycle of P450, Compound I is believed to be a Fe^IV^ oxyferryl species with two unpaired electrons in an iron-oxo moiety and a third unpaired electron in a porphyrin. The theoretical and experimental studies indicated that the propionate side chains are crucial in determining the nature of the third unpaired electron [19, 20]. Additionally, these results proposed a pathway involving the propionate groups for the intramolecular electron transfer and inducing the delocalization of the spin density in the high-valent metal-oxo species [21].

**Scheme 2:**
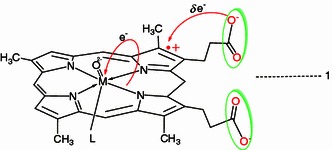
The electron transfer from the porphyrin system into the metal center and from the propionate lone pairs into the porphyrin system

Furthermore, the active role of the hemin propionate groups in ligand binding has also been observed and the electron transfer (spin delocalization) from the porphyrin system into the metal center and from the propionate lone pairs into the porphyrin system also have been suggested (Scheme 2).

If we change the propionate by introduction of electronic unsaturated substituents, the propionate lone pairs might be perturbed in a predicted fashion, activating an electron delivery pathway from the propionate into the porphyrin π orbitals and the metal center, but in light of the current data any suggestion would be merely speculative. On the other hand, the electron-donor groups at the propionate side chains were catalytically more efficient on the toluene hydroxylation than their bearing the electron-withdrawing groups. The best conversion and yield of benzyl alcohol were obtained in the presence of DP(–SS–) and Co-DP(–NH_2_)_2_. These results are presumably due to the unpaired electrons of functional groups. Especially, the Co-DP(–SS–) can be considered as the best model for mimic the cytochrome P450 used in various reactions.

## Conclusions

A series of cobalt porphyrins was readily prepared by modification of propionate side chains and these metalloporphyrin complexes were found to act as efficient catalysts in the oxidation of toluene to benzaldehyde and benzyl alcohol using air as the oxygen source. We have found that the presence of different groups at the deuteroporphyrin-propionate side chains is capable of modifying the yield and selectivity of the investigated reaction as well as the stability of the catalysts. It is worth noting also that the electron-donor groups attached to the propionate side chains showed higher catalytic efficiencies than the electron-withdrawing ones in the oxidation of toluene. Therefore, the modification of propionate side chains should be considered with caution on the design of metalloporphyrin-based catalysts, particularly those inspired in P450 like structure.
